# A new species of *Chalicodoma* from Saudi Arabia with modified facial setae (Hymenoptera, Megachilidae)

**DOI:** 10.3897/zookeys.204.3228

**Published:** 2012-06-25

**Authors:** Abdulaziz S. Alqarni, Mohammed A. Hannan, Victor H. Gonzalez, Michael S. Engel

**Affiliations:** 1 Department of Plant Protection, College of Food and Agriculture Sciences, King Saud University, P.O. Box 2460, Riyadh 11451, Kingdom of Saudi Arabia; 2 Division of Entomology, Natural History Museum, and Department of Ecology & Evolutionary Biology, 1501 Crestline Drive – Suite 140, University of Kansas, Lawrence, Kansas 66045, USA

**Keywords:** Apoidea, Anthophila, bees, *Blepharis ciliaris*, Megachilinae, Megachilini, taxonomy, nototribic flowers

## Abstract

Some bees and pollen wasps have independently evolved simple, stiff, erect, apically-curved, curly or hooked facial setae as adaptations to collect pollen from nototribic flowers. A distinctive new species of *Chalicodoma* Lepeletier de Saint Fargeau subgenus *Pseudomegachile* Friese from Saudi Arabia with such morphological adaptations, *Chalicodoma riyadhense* **sp. n.**, is described and figured. The species was captured visiting flowers of *Blepharis ciliaris* (L.) (Acanthaceae). The occurrence of modified facial setae is documented and discussed for the first time in eight other species of *Pseudomegachile*, and a key to the genera and subgenera of Megachilini currently confirmed for Saudi Arabia is provided.

## Introduction

The presence of erect or proclinate, stiff, apically curly or hooked setae on certain areas of the face of some bees and pollen wasps (Vespidae: Masarinae) is related to pollen-collecting behavior from nototribic flowers, or bilateral flowers in which filaments and styles are located on the adaxial side or top of the flower, particularly in the families Lamiaceae, Fabaceae, and Plantaginaceae (Müller 1996; Thorp 2000; Michener 2007). In some species, such as *Anthophora walteri* Gonzalez and *Anthidium rodriguezi* Cockerell, the areas of the face covered by these setae are also sometimes distinctly flat to nearly depressed, with the integument dull, coarsely and sparsely punctate (Brooks 1988; Michener et al. 2003; Gonzalez and Chavez 2004; Gonzalez and Griswold in prep.). Such morphological adaptations occur across multiple genera from different families (e.g., Thorp 2000; Michener 2007; Rightmyer et al. 2011). The modified setae vary among taxa in terms of the shape, length, thickness, orientation, and distribution. In some species they are short and erect and are found on the entire face (e.g., *Osmia calaminthae* Rightmyer et al.), in others they are rather long, apically curved or cork-screw shaped, and are present on the clypeus and supraclypeal area only; they can also be proclinate and form a basket on the vertex and frons, as in *Osmia brevis* Cresson (Tepedino et al. 1999; Rightmyer et al. 2011). In several *Anthophora* Latreille, in addition to the apically curved setae, there is a distinct row of stout, blunt setae across the base of the clypeus known as the pecten (Brooks 1988). Such a variation may indicate degrees of specialization on host plants, but observations are lacking.

Little is known about the foraging behavior of these bees on nototribic flowers but scant observations suggest some behavioral adaptations in how they harvest the pollen from the flower and remove it from the body, especially for those that exhibit modifications on both the facial setae and the integument (e.g., Müller 1996; Gonzalez et al. 2006). Although some of these morphological variations as well as the foraging behavior of bees were described in detail by Müller (1996) for 13 central European species (seven genera and three families), and have been sporadically mentioned in the literature from largely anecdotal observations, they remain to be thoroughly documented and studied across the Apoidea.

In this paper, we describe a distinctive new species of *Chalicodoma* Lepeletier de Saint Fargeau subgenus *Pseudomegachile* Friese from Saudi Arabia with modified facial setae. To date, the only other known *Chalicodoma* having similar setae is *Chalicodoma (Chalicodoma) albocristata* (Smith) from the western Palearctic (Müller 1996). Also, based on the study of specimens from more than half of the species-diversity of the subgenus, we document and discuss the occurrence of modified facial setae in eight other species of *Pseudomegachile*. Herein, *Chalicodoma* is recognized in a narrower sense than that of Michener (1962, 1965), and presently includes eight subgenera (*Alocanthedon* Engel and Gonzalez, *Callomegachile* Michener, *Cestella* Pasteels, *Chalicodoma* s.str., *Cuspidella* Pasteels, *Gronoceras* Cockerell, *Largella* Pasteels, and *Pseudomegachile*). *Pseudomegachile* is as highly diverse and morphologically heterogeneous as *Callomegachile*. It is widespread in the Eastern Hemisphere, across the Palearctic and Oriental regions but *Callomegachile lanata* (Fabricius) has been introduced to Florida, USA and the West Indies and *Callomegachile ericetorum* (Lepeletier de Saint Fargeau) to Canada (Michener 2007; Sheffield et al. 2010).

## Material and methods

Material considered herein is deposited in the King Saud University Museum of Arthropods, Plant Protection Department, College of Food and Agriculture Sciences, King Saud University, Riyadh, Kingdom of Saudi Arabia (KSMA) and Division of Entomology (Snow Entomological Collections), University of Kansas Natural History Museum, Lawrence, Kansas, USA (SEMC). Morphological terminology follows that of Engel (2001) and Michener (2007), except for torulus herein used instead of antennal alveolus, while the format for the description is largely based on that of Gonzalez et al. (2010) and Engel and Gonzalez (2011). Photomicrographs were prepared using a Canon 7D digital camera attached to an Infinity K-2 long-distance microscope lens. Measurements were made with an ocular micrometer attached to an Olympus SZX-12 stereomicroscope. To explore and document the occurrence of modified facial setae in *Pseudomegachile*, we examined specimens of 43 species deposited in SEMC that occur across the distribution range of the subgenus.

## Systematics

### Genus *Chalicodoma* Lepeletier de Saint Fargeau. Subgenus *Pseudomegachile* Friese

#### Chalicodoma (Pseudomegachile) riyadhense sp. n.

urn:lsid:zoobank.org:act:FBCBB044-B2EA-47AC-BC4B-D68EFAE56B9C

http://species-id.net/wiki/Chalicodoma_riyadhense

Figs 1 –17

##### Holotype.

♀, Saudi Arabia, Riyadh, Al Amariah, [Mazra’ah] Majra Al-Gasim [farm], 23-v-2011 [23 May 2011], M.A. Hannan // at flowers of *Blepharis ciliaris* [(L.) Acanthaceae] (KSMA).

##### Paratypes.

1♂, 1♀, with the same data as the holotype (SEMC); 2♂♂, same data as holotype except 31-v-2011 [31 May 2011], I. Naser // at *Blepharis ciliaris* (SEMC & KSMA); 1♂, same data as holotype (KSMA); 1♂, same data as holotype except 17-v-2011 [17 May 2011] (KSMA).

##### Diagnosis.

**Figures 1–6.**
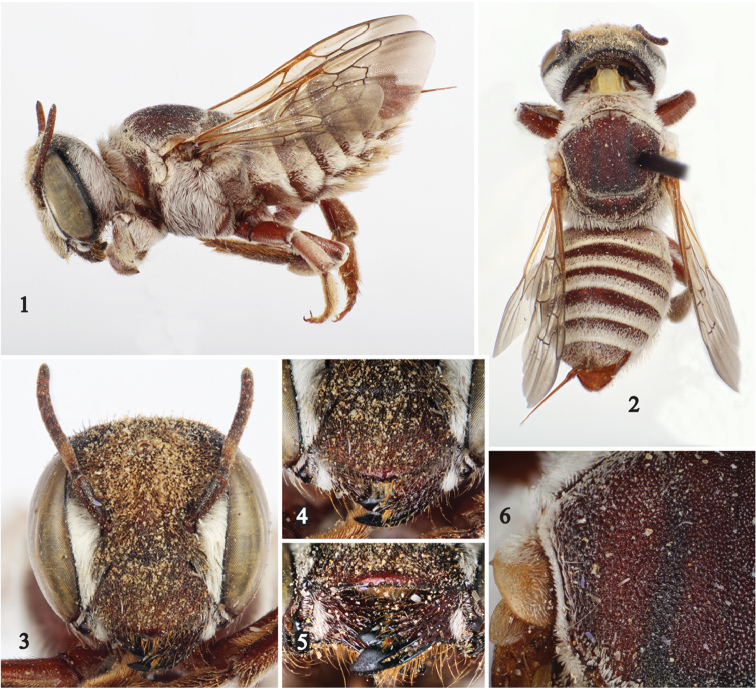
Photomicrographs of female holotype (KSMA) of *Chalicodoma (Pseudomegachile) riyadhense* sp. n. **1** Lateral habitus **2** Dorsal habitus **3** Facial view **4** Detail of clypeus **5** Detail of mandibles **6** Detail of mesoscutal integument.

**Figures 7–17.**
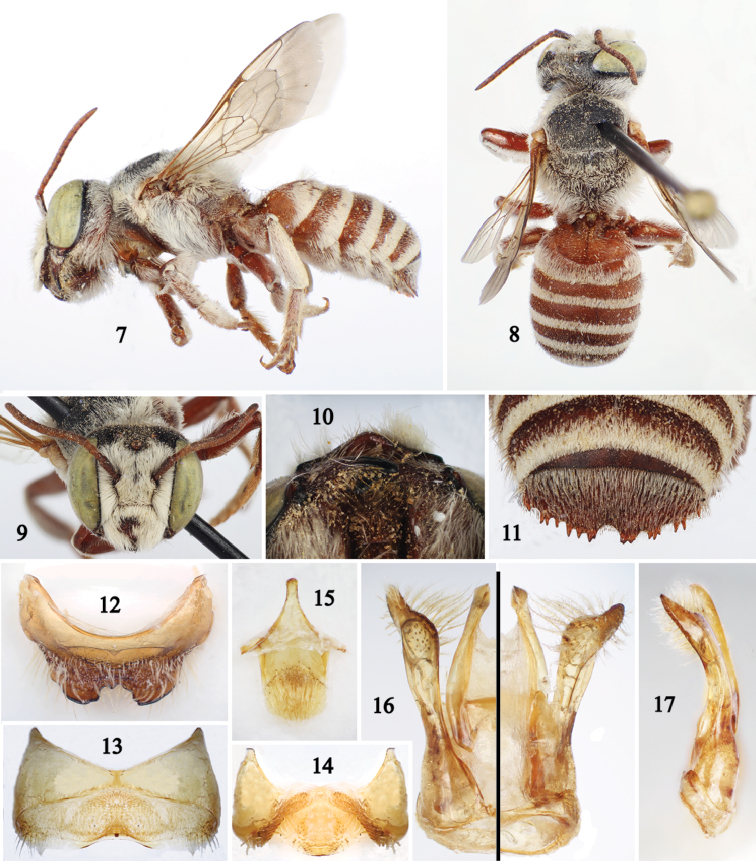
Photomicrographs of male paratype (SEMC) of *Chalicodoma (Pseudomegachile) riyadhense* sp. n. **7** Lateral habitus **8** Dorsal habitus **9** Facial view **10** Ventral view of head **11** Apex of sixth metasomal tergum **12** Seventh tergum **13** Fifth metasomal sternum **14** Sixth sternum **15** Eighth sternum **16** Genital capsule (dorsal left, ventral right) **17** Lateral aspect of genital capsule.

The female of this species can be recognized easily by the following combination of characters: body light reddish brown contrasting with dense, minutely-branched white setae on the body (Figs 1, 2); preoccipital border distinctly concave in dorsal view; clypeus, supraclypeal area, frons, and upper paraocular area with modified facial setae; and clypeus with distal margin distinctly impunctate and swollen medially (Figs 4, 5). In addition to the body coloration and pubescence (Figs 7, 8), which are similar to those of the female, the male can be recognized by the following combination of characters: clypeus distinctly pointed on distal margin (Fig. 10); mandible without inferior process; tarsi of all legs unmodified but with small dark spot on inner surface of probasitarsus; sixth metasomal tergum horizontal in profile, and seventh tergum with strong, transverse preapical carina deeply notched medially (Fig. 12).

The reddish body coloration contrasting with the white dense pubescence of *Chalicodoma riyadhense* resemble some Palearctic and Oriental species, such as *Chalicodoma flavipes* (Spinola), *Chalicodoma rubripes* (Morawitz), and *Chalicodoma xanthocneme* (Alfken), but it is easily separated from those and any other known species of *Pseudomegachile*, including those with modified facial setae, by the distinctive clypeus in both sexes. Furthermore, the male of *Chalicodoma riyadhense* is also unique in having a seventh metasomal tergum with strong, medially notched preapical carina. Except for some species, such as *Chalicodoma kigonserana* (Friese) and *Chalicodoma nigrocaudata* (Friese), most *Pseudomegachile* males have a seventh tergum with a complete or medially pointed preapical carina. However, those males with similar bilobed preapical carina are much larger in size (18‒20 mm in body length), have very long procoxal spines, strongly modified protarsi, and a different color pattern of the pubescence (head and mesosoma covered with black setae contrasting with ferruginous setae on the metasoma).

##### Description.

*Female*. Body length 10.4 mm; forewing length (measured from apex of humeral sclerite to wing margin) 6.4 mm. Head 1.1 times broader than long; inner orbits of compound eyes parallel-sided; intertorular distance 2.2 times torulorbital distance; interocellar distance 3.2 times median ocellar diameter, 1.7 times ocellocular distance; ocelloccipital distance 1.7 times median ocellar diameter; torular process reduced, barely visible; vertex rounded in frontal view (Fig. 3); preoccipital border rounded, distinctly concave in dorsal view (Fig. 2); compound eye about 2.3 times longer than wide; gena slightly narrower (0.8x) than compound eye in profile, narrowest dorsally; mandible with four teeth, apical two longer and broader than basal two (Fig. 5); clypeus about 1.7 times broader than long, with epistomal sulcus gently convex basally, well projected over clypeal-labral articulation, elevated along midline on disc, distinctly swollen on distal margin medially (Figs 4, 5); supraclypeal area slightly elevated along midline; frons convex in profile, not flat; scape about 3 times longer than broad, not reaching lower margin of median ocellus in repose, pedicel about as long as broad, first flagellomere about as long as broad, about equal to pedicel length, 1.5 times longer than second flagellomere, subsequent flagellomeres progressively increasing in length towards apical flagellomeres, distalmost flagellomere longest. Omaulus rounded; pronotal lobe carinate; mesoscutum gently convex in profile; mesoscutellum flat on disc; pretarsal claws simple, basally with two unmodified simple setae, basal seta shortest; pro- and mesotibiae distally with angled medial projection on outer surfaces, not forming distinct spinose process. Second to fifth metasomal terga with elevated discal areas (i.e., premarginal lines distinct); sixth tergum straight in profile.

Body color light reddish brown except dark brown to black as follows: mandible distally, clypeal margin, epistomal sulcus, vertex, antenna (darker on outer surfaces of scape and pedicel), and mesoscutum and mesocutellum with faint spots on discs (Figs 2, 6). Tegula and humeral sclerite yellowish; wings hyaline, darker distally, veins yellowish basally (including pterostigma), dark brown distally.

Integument smooth and shiny between punctures except strongly imbricate on basal area of propodeum and weakly imbricate on metasomal sterna and terga. Face finely and closely punctate, nearly contiguous except on distal margin of clypeus medially smooth and shiny, punctures smaller on supraclypeal area; gena with shallower punctures than on face. Mesoscutum and mesoscutellum with punctures coarser and larger than on head (Fig. 6), those on mesoscutellum larger than on mesoscutum; mesepisternum with larger and coarser punctures than on metepisternum and sides of propodeum, punctures shallow on metepisternum; basal area of propodeum weakly striate basally. Metasomal terga finely punctate, punctures not contiguous, smaller than on mesoscutum; sterna with punctures coarser, sparser than on terga.

Pubescence white, unless indicated otherwise. Following areas with dense (integument obscured or barely visible among setae), minutely-branched, appressed or semierect setae: mandible basally, lower and middle paraocular areas, gena, pronotum, pronotal lobe, anterior half of tegula, humeral sclerite, margins of mesoscutum, mesoscutellum, metanotum, propodeum, remaining areas of mesosoma laterally, coxae, trochanters, posterior surfaces of pro- and mesofemora, anterior surface of metatibia, outer surfaces of tibiae, outer surfaces of pro- and mesobasitarsi, first metasomal tergum entirely, depressed marginal areas of second to fourth terga (apical fasciae), fifth tergum entirely, distal margin of first sternum, sides of second to fourth sterna, and entire distal margin of fifth sternum; setae longer on head and mesosoma than on metasoma (Figs 1, 2). Clypeus, supraclypeal area, frons, and upper paraocular area with simple, stiff, apically-curved yellowish setae (at most as long as median ocellar diameter) sparsely covering integument (Fig. 3). Lower margin of mandible and gena inferiorly with long (about two times median ocellar diameter), erect, yellowish simple setae. Second to sixth metasomal sterna with yellowish scopal setae, shorter on sixth sternum.

*Male*. As described for female except for usual secondary sexual characters and following: Body length 11.6 mm; forewing length 6.4 mm. Head 1.2 times broader than long; inner orbits of compound eyes slightly converging below; intertorular distance 1.8 times torulorbital distance; interocellar distance 2.1 times median ocellar diameter, 1.8 times ocellocular distance; ocelloccipital distance 1.4 times median ocellar diameter; vertex slightly flat medially in frontal view (Fig. 9); preoccipital border not as distinctly concave as in female; compound eye about 2.6 times longer than wide; mandible with four teeth, without inferior process; clypeus more convex than in female, with distal margin distinctly projecting over clypeal-labral articulation, so that triangular in ventral view; scape 2.7 times longer than broad, reaching lower margin of median ocellus in repose, second to eleventh flagellomeres longer than broad. Procoxal spine small, about half length of median ocellar diameter as measured in profile; tarsi of all legs unmodified, but probasitarsus with inner surface asetose, with small dark spot; pretarsal claws cleft, inner ramus shorter than outer. Sixth metasomal tergum horizontal or nearly so in profile, without lateral projection on distal margin, transverse preapical carina strong, irregularly toothed, with broad median emargination (Fig. 11); seventh tergum with transverse preapical carina strongly projected, with deep median emargination thus forming a distinct lateral lobe (Fig. 12); fifth to eighth sterna (seventh sternum obsolescent in Megachilini) and genital capsule as in figures 13–17; volsella with apex distinctly notched or bilobed.

Body color slightly lighter than female but with frons, vertex, gena dorsally, mesoscutum, and mesoscutellum black (Figs 7, 8).

Pubescence in general longer and denser than female, particularly on discs of mesoscutum, mesoscutellum, and metasomal terga; clypeus, supraclypeal area, paraocular area, and frons densely covered by setae (integument obscured); procoxa with dense patch of short ferruginous setae above spine, coxa otherwise sparsely covered with branched setae. Fifth metasomal sternum with spatulate setae midbasally on postgradular area, setae otherwise minutely branched, except apicolaterally with long, simple setae; sixth sternum with dense spatulate setae basolaterally on postgradular area forming distinctive patch, otherwise setae apically flattened and curved; sternum eight without setae on or near lateral margin but ventrally with dense, minutely-branched setae on disc (Fig. 15).

##### Etymology.

The specific epithet refers to the greater Riyadh area in Saudi Arabia, and from where the species was collected.

##### Host plant.

**Figures 18–20.**
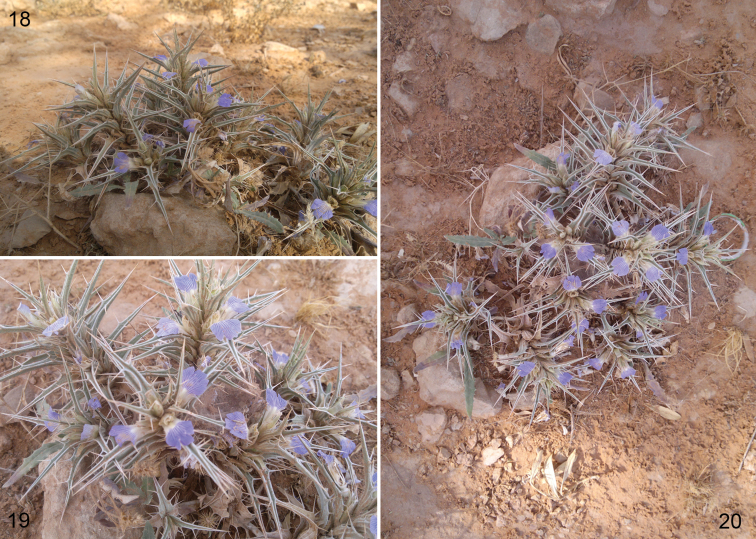
Photographs of *Blepharis ciliaris* (L.) (Acanthaceae) in Saudi Arabia. **18** Entire plant in lateral view **19** Detail of flowers **20** Entire plant as viewed from above (photographs by A.S. Alqarni).

The species was captured visiting flowers of *Blepharis ciliaris* (L.) (Acanthaceae), locally known as “Saha” or “Naqie” (Figs 18–20). The plant is a perennial herb, usually about 20 cm in height (10–30 cm), growing in small patches with small blue to light violet flowers and easily found in the area of Al Amariah, approximately 25 km northwest of Riyadh, from early April through early June. The species prefers the silt bottoms of rocky wadi basins. *Blepharis ciliaris* is a good source of nectar, particularly in the southwest and in years following a good rainy season. Regional beekeepers sometimes produce “Saha honey”.

### Key to genera and subgenera of Megachilini of Saudi Arabia

Records of the occurrence of several megachiline species in Saudi Arabia remain to be confirmed and a key to species is therefore not presented at this time. The following key, which is modified from Michener (2007), will assist in the identification of the megachiline genera and subgenera that are presently confirmed to occur in Saudi Arabia.

**Table:** 

1	Females	2
–	Males	6
2(1)	Scopa absent; metasoma tapering from near base to narrow, apex often acutely pointed; cleptoparasite	*Coelioxys (Allocoelioxys)*
–	Scopa present on metasomal sterna two through five or six; metasoma not distinctly tapering throughout its length; free living	3
3(2)	Sixth metasomal sternum with a fringe of branched setae on or near apical margin; mandible with cutting edge in at least one interspace, sometimes hidden behind margin of interspace	4
–	Sixth metasomal sternum without apical fringe of branched setae; mandible without cutting edges between teeth	5
4(3)	Mandible five- or six-toothed, teeth (except first) similar and with similarly-shaped, incomplete cutting edges in second and third (and sometimes fourth) interspaces; apices of mandibular teeth roughly equidistant from nearest neighbors; preapical transverse mandibular groove distinct and filled with short, fine, pale setae	*Megachile (Creightonella)*
–	Mandible four- to five-toothed, teeth above first of different shapes and cutting edges often of different shapes; apices of mandibular teeth commonly separated from nearest neighbors by different distances; preapical transverse mandibular groove, if present, not filled with short, pale setae	*Megachile (Eutricharaea)*
5(3)	Distal margin of clypeus irregularly rounded (rarely weakly emarginate medially), usually strongly crenulate, produced well over base of labrum, not thickened; mandible usually slender with apical margin strongly oblique; head little developed posteriorly, ocelloccipital distance thus not greater than interocellar distance	*Chalicodoma (Chalicodoma)*
–	Distal margin of clypeus truncate, not crenulate, often not much produced over base of labrum, but if rounded and somewhat crenulate, then margin thickened and impunctate; head usually much developed posteriorly, ocelloccipital distance thus greater than interocellar distance	*Chalicodoma (Pseudomegachile)*
6(1)	Sixth metasomal tergum with multispinose preapical carina, with two pairs of long, preapical spines, each spine of upper pair sometimes divided into two, or crenulate, rounded, or fused to other spine of pair; cleptoparasite	*Coelioxys (Allocoelioxys)*
–	Sixth metasomal tergum with preapical carina not as above, often crenulate, medially emarginate, or sometimes reduced to two spines; free living	7
7(6)	Fifth and sometimes sixth metasomal sterna exposed and generally similar to preceding sterna (sometimes fifth sternum largely hidden but sixth sternum exposed); lateral extremity of carina of sixth tergum directed basad, away from apical margin of tergum	*Megachile (Creightonella)*
–	Fifth and sixth metasomal sterna retracted, variously modified, less sclerotized, less punctate, and less setose than first to fourth sterna; lateral extremity of carina of sixth tergum absent or directed toward lateral extremity of apical margin of tergum	8
8(7)	Eighth metasomal sternum without marginal setae but discal setae sometimes extending beyond margin laterally; metasoma usually less strongly convex and usually less than twice as long as wide	*Megachile (Eutricharaea)*
–	Eighth metasomal sternum *often* with lateral marginal setae; metasoma commonly strongly convex and twice as long as wide or more	9
9(8)	Toothed margin of mandible (three- to four-toothed) strongly oblique, nearly as long as distance from upper tooth to mandibular base; seventh metasomal tergum with narrow, median, apically-truncate projection extending well beyond teeth of sixth tergal carina; apex of gonostylus asetose or with very short, poorly-branched to simple setae	*Chalicodoma (Chalicodoma)*
–	Toothed margin of mandible (three-toothed) less oblique, much shorter than distance from upper tooth to mandibular base; seventh metasomal tergum a low sclerite largely hidden behind sixth tergum, sometimes produced to small median spine; apex of gonostylus often with long, densely-branched setae on medial margin	*Chalicodoma (Pseudomegachile)*

## Discussion

The bee fauna of Saudi Arabia is rich but relatively little known biologically and taxonomically. Already several new species and new records are accumulating from the material collected by the authors (e.g., Alqarni et al. 2012; Engel et al. in press), and further accounts of this fauna will be forthcoming (ASA, MAH, MSE in prep., unpubl. data). Thus, the new species described herein is a small contribution toward this larger effort to elucidate the Arabian melittological fauna.

Including the species described herein, we found nine species of *Pseudomegachile* with modified facial setae (Table 1). Hitherto, the only known species of *Chalicodoma* with modified facial setae was *Chalicodoma albocristata* (Müller 1996). In all *Pseudomegachile* species, the modified setae consisted of simple, stiff, erect or proclinate, apically-curved setae. They were often found on the clypeus and supraclypeal area, and in some species also on the frons, as is the case for *Chalicodoma riyadhense*. No modifications of the integument or in the areas of the face covered by these setae were observed. That is, in all species the integument was shiny and densely punctate, and the clypeus, supraclypeal area, and frons were not distinctly flat or depressed. Although we examined more than half of the known species of *Pseudomegachile*, these observations are doubtless preliminary. The desert plant species on which the new bee species was collected, *Blepharis ciliaris*, has nototribic flowers, thus agreeing with the modified facial setae found on *Chalicodoma riyadhense*. This suggests that *Chalicodoma riyadhense* may be a regular visitor of this plant but it would be interesting to know to what extent it contributes to the pollination of *Blepharis ciliaris* and how much it specializes on it. However, we hope to draw more attention to and encourage melittologists to document these morphological adaptations as well as the floral associations and foraging behavior of the bees that have them.

**Table 1.** Species of *Chalicodoma* Lepeletier de Saint Fargeau subgenus *Pseudomegachile* Friese with modified facial setae for pollen collection from nototribic flowers. Modified setae refer to simple, erect or proclinate, apically curved setae. Plus (+) and dash (–) symbols indicate presence or absence of a character; * = not distinctly modified. In *Chalicodoma riyadhense* sp. n. the modified setae are also found on the upper paraocular area. The locality corresponds to the areas where the examined specimens were collected, not the total distribution of those species. It must be noted that *Chalicodoma* (from Gr. χάλιξ, χάλιχος, calx + δωμα, domus) is of neuter gender, and all names now referred to that genus have been made to conform. The derivation of the name was indicated by Brullé (1832), who gave: “*nom tiré de l’habitude de l’insecte, qui construit son nid avec de petits cailloux, comme Réaumur nous l’a fait connaître dans ses intéressants mémoires*”. However, only those names which are latinized adjectival or participial species-group names are adjusted in gender (ICZN 1999: Art. 34.2), which means that the forms “angonicum”, “farinosum”, and “kigonseranum” which have appeared in various sources are all improper emendations.<br/>

**Species**	**Modified setae**			**Locality**
	**Clypeus**	**Supraclypeal area**	**Frons**	
*Chalicodoma angonica* (Cockerell)^1^	+	+	+	Zambia [N.E. Rhodesia]
*Chalicodoma cinnamomeum* (Alfken)	+	–	–	Morocco, United Arab Emirates
*Chalicodoma farinosa* (Smith)	+	+	–	Greece
*Chalicodoma kigonserana* (Friese)	+	–	–	Tanganyika, Democratic Republic of Congo [Belgian Congo], Zambia [N. Rhodesia], Malawi
*Chalicodoma lualabae* (Cockerell)^2^	+	+	+	Zambia [N.E. Rhodesia]
*Chalicodoma marshalli* (Friese)	+	+	+	Tanganyika, Zambia [N. Rhodesia]
*Chalicodoma riyadhense* sp. n.	+	+	+	Saudi Arabia
*Chalicodoma seraxense* (Radoszkowski)	+	+	+	S. India
*Chalicodoma transgrediens* (Rebmann)	+	+*	–	Turkey

^1^ Note that *Chalicodoma angonica* was erroneously synonymized by Pasteels (1965) with *Chalicodoma kigonserana*.

^2^ Note that this species is possibly a junior synonym of *Chalicodoma angonica*.

## Supplementary Material

XML Treatment for Chalicodoma (Pseudomegachile) riyadhense
